# Malignant epithelioid hemangioendothelioma of the ear

**DOI:** 10.1093/jscr/rjac062

**Published:** 2022-03-09

**Authors:** Radha Senaratne, Máire-Caitlín Casey, Jack L Kelly

**Affiliations:** Department of Plastic & Reconstructive Surgery, Galway University Hospital, Galway, Ireland; Department of Surgery, National University of Ireland, Galway, Ireland; Department of Plastic & Reconstructive Surgery, Galway University Hospital, Galway, Ireland; Department of Plastic & Reconstructive Surgery, Galway University Hospital, Galway, Ireland

**Keywords:** skin, cancer, plastic surgery, flap, epithelioid hemangioendothelioma

## Abstract

Skin cancer is the most common cancer in Ireland. Our patient presented for removal of a cutaneous lesion on his ear. The histopathological diagnosis was malignant epithelioid hemangioendothelioma. It is very rare for this to present primarily as a cutaneous lesion. Here, we discuss the management of this patient and the surveillance he required. It is important to consider alternative histological diagnoses in patients presenting with cutaneous lesions and how this affects management and prognosis.

## INTRODUCTION

Skin cancer is the most common cancer in Ireland, with 11 000 new cases diagnosed each year. Nonmelanoma skin cancer (NMSC) accounts for >90% of skin cancer diagnoses [1]. The ear is the fifth most common site for NMSC on the head [2]. The structure of the ear makes it a challenge to reconstruct, and lesions are often incompletely excised [3]. NMSC presenting on the ear tends to behave more aggressively than other anatomical locations and therefore requires definitive management and close surveillance [3].

Malignant epithelioid hemangioendothelioma (EH) is a tumor of vascular origin, which is exceptionally rare, and it is highly unusual for it to present as a cutaneous lesion. This case report outlines the diagnosis and management of a patient who presented with a lesion on his pinna and was post-operatively diagnosed with EH.

## CASE REPORT

**Figure 1:**
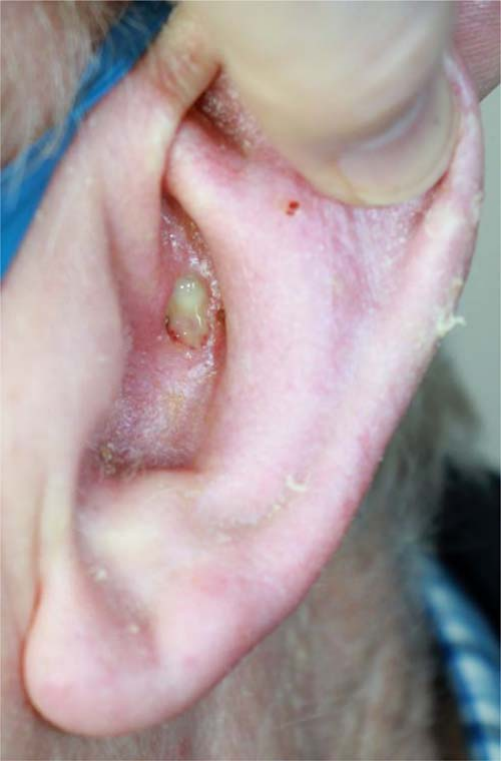
Ulcerated lesion on left ear prior to excision.

An 80-year-old gentleman was referred by his general practitioner with a lesion on his left ear (Fig. 1). He underwent an excisional biopsy and reconstruction with a trap door flap without complication.

**Figure 2:**
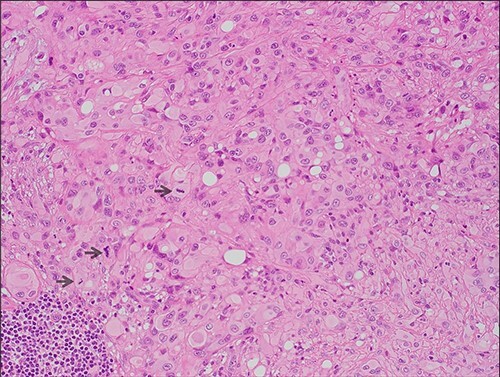
Microscopic features (x20, H&E) of the lesion which comprises of sheets of epithelioid cells with eosinophilic hyaline cytoplasm; intracytoplasmic lumina and focal pleomorphism are evident; mitotic figures are highlighted by arrows.

The excised specimen revealed a 13-mm tumor which invaded down to elastic cartilage with ulceration and focal necrosis. The tumor was composed of sheets of epithelioid cells with eosinophilic hyaline cytoplasm and intracytoplasmic vacuoles (Fig. 2).

The mitotic index was up to 6/10 high-powered fields (HPF). No lymphovascular invasion was observed. The peripheral margin was 1.9 mm and the deep margin was 2 mm. Immunohistochemistry showed positivity for CD31 and the tumor cells were strongly positive for ERG. The pathologic features supported a diagnosis of malignant EH.

**Figure 3:**
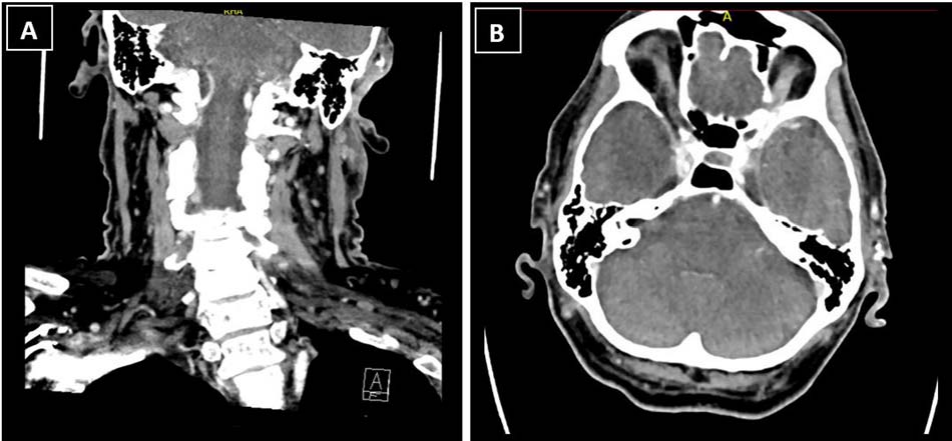
CT neck showed a 1.3 × 1.2 × 1.3-cm ill-defined soft tissue enhancement above the left external auditory canal involving the subcutaneous tissue and overlying skin; (**A**) coronal view; (**B**) axial view.

The patient’s case was discussed at the skin cancer multidisciplinary team meeting and he underwent imaging of the area for further evaluation, given the histological diagnosis. Computed tomography (CT) neck showed a 1.3 × 1.2 × 1.3-cm ill-defined soft tissue enhancement above the left external auditory canal involving the subcutaneous tissue and overlying skin (Fig. 3). There was involvement of the superior and superficial aspect of the parotid gland. CT thorax, abdomen and pelvis showed no evidence of distant metastases.

The patient underwent further wide local excision which revealed negative margins. Positron emission tomography CT after re-excision of margins showed no FDG avid areas, indicating no remaining disease. The patient went on to have adjuvant radiotherapy.

## DISCUSSION

Malignant EH is a rare neoplasm of vascular origin. It can present in a wide variety of anatomical locations; however, it most commonly affects the liver, lungs and bones. Its prevalence is approximately one in a million globally [4].

The histological and immunohistochemical findings demonstrated in this case are typical of EH. The microscopic features of epithelioid cells with eosinophic hyaline cytoplasm and intracytoplasmic vacuoles are common features of EH [5]. Both ERG and CD31 are highly sensitive for EH, and one case series [5] documented 100% of cases staining positive for these markers.

Deyrup *et al*. [6] proposed risk stratification criteria for EH and reported that tumors >3 cm and with a mitotic index >3/10 HPF tend to be more aggressive and have a poorer prognosis. The mitotic index of up to 6/10 in this case demonstrates that this tumor is potentially high risk.

When EH presents elsewhere, its appearance in visceral organs can mistakenly lead to a wide range of differential diagnoses. It is exceptionally rare for EH to occur on the skin, with few reported cases [7–10]. Our patient presented with a non-healing ulcer on his ear (Fig. 1). There has been one other case documented in the literature in which the patient presented with such a finding [11]. The patient in that case had metastatic disease in one lymph node and therefore required neck dissection as well as total auriculectomy with adjuvant radiotherapy. This demonstrates that EH has the potential to spread to surrounding lymph nodes, so requires aggressive initial management as well as close follow-up surveillance.

The ear is one of the most anatomically challenging areas to reconstruct. The location of the lesion on the patients concha limits reconstructive options. In this case, options for reconstruction include a full thickness skin graft or a local flap.

A full thickness skin graft involves a skin graft taken from a donor site such as the pre-auricular area, the donor site closed primarily and the graft inset over the defect. The limitation of this option is that is does not reconstruct the cartilage.

The trap door flap provides a reliable way to reconstruct conchal defects. This flap was first described in 1972 by Masson [12]. It is raised on the mastoid and post-auricular surfaces and its blood supply is based off the post-auricular vessels. The donor site is closed primarily. This flap gives an excellent cosmetic result and the donor site scar is well hidden. The trap door flap is an excellent and reliable choice for reconstruction of the concha [13] and was the reconstructive method used in this case. The patient had a satisfactory post-operative result.

Due to the high-risk nature of the lesion, the patient had a follow-up CT neck 3 months after completion of radiotherapy. He will continue to undergo close surveillance with clinical examination and imaging.

## CONCLUSION

This case demonstrates a rare neoplasm which unusually presented as a cutaneous lesion. The location of the lesion on the ear is a challenging area for the surgeon to reconstruct. Local flaps, such as the trap door flap as used in this case, provide adequate reconstruction and are cosmetically acceptable. Malignant EH requires aggressive management, given its potential to metastasize to lymph nodes, and it requires continued surveillance after treatment. It is important to consider alternative histological diagnoses in patients presenting with cutaneous lesions and how this affects management and prognosis.

## CONFLICT OF INTEREST STATEMENT

None declared.

## FUNDING

None.

## CONSENT STATEMENT

Informed consent was obtained from the patient for publication of this article.
