# Correction to: Malignant epithelioid hemangioendothelioma of the ear

**DOI:** 10.1093/jscr/rjac207

**Published:** 2022-05-31

This is a correction to: Radha Senaratne, Máire-Caitlín Casey, Jack L. Kelly, Malignant epithelioid hemangioendothelioma of the ear, *Journal of Surgical Case Reports*, Volume 2022, Issue 3, March 2022, rjac062, https://doi.org/10.1093/jscr/rjac062.

In the originally published version of this manuscript, the corresponding author contact telephone number was incorrect.

This error has been corrected.

